# Dynamic distribution and tissue tropism of *classical swine fever virus *in experimentally infected pigs

**DOI:** 10.1186/1743-422X-8-201

**Published:** 2011-05-02

**Authors:** Jun Liu, Xue-Zheng Fan, Qin Wang, Lu Xu, Qi-Zu Zhao, Wei Huang, Yuan-Cheng Zhou, Bo Tang, Lei Chen, Xing-Qi Zou, Sha Sha, Yuan-Yuan Zhu

**Affiliations:** 1 China Institute of Veterinary Drug Control, National Classical Swine Fever Reference Laboratory, Beijing 100081, China; 2 Liao Ning Institute of Animal Health Inspection, Shenyang 110015, China; 3 South West University, Rongchang, Chongqing 402460, China

## Abstract

**Background:**

*Classical swine fever *(CSF), caused by the *Classical swine fever virus *(CSFV), is an Office International des Epizooties (OIE) notifiable disease. However, we are far from fully understand the distribution, tissue tropism, pathogenesis, replication and excretion of CSFV in pigs. In this report, we investigated the dynamic distribution and tissue tropism of the virus in internal organs of the experimentally infected pigs using real-time RT-PCR and immunohistochemistry (IHC).

**Results:**

A relative quantification real-time PCR was established and used to detect the virus load in internal organs of the experimentally infected pigs. The study revealed that the virus was detected in all 21 of the internal organs and blood collected from pigs at day 1 to day 8 post infections, and had an increasing virus load from day 1 to day 8 post infections. However, there was irregular distribution virus load in most internal organs over the first 2 days post infection. Blood, lymphoid tissue, pancreas and ileum usually contain the highest viral loads, while heart, duodenum and brain show relatively low viral loads.

**Conclusions:**

All the data suggest that CSFV had an increasing virus load from day 1 to day 8 post infections in experimentally infected pigs detected by real-time RT-PCR, which was in consistent with the result of the IHC staining. The data also show that CSFV was likely to reproduce in blood, lymphoid tissue, pancreas and the ileum, while unlikely to replicate in the heart, duodenum and brain. The results provide a foundation for further clarification of the pathogenic mechanism of CSFV in internal organs, and indicate that blood, lymphoid tissue, pancreas and ileum may be preferred sites of acute infection.

## Background

*Classical swine fever *(CSF) is a highly contagious and often fatal disease of swine, affecting domestic and wild boar populations. *Classical swine fever virus *(CSFV), the causative agent of CSF, is a member of the genus *Pestivirus*, which belongs to the *Flaviviridae *family [1]. Other important animal pathogens within the genus *Pestivirus *are *bovine viral diarrhea virus *(BVDV) that affects cattle and *Border disease virus *(BDV) that affects sheep; both of which can naturally infect pigs.

CSFV is an enveloped virus with about a 12.3-kb single-stranded RNA genome of positive polarity. The genome contains only a single open reading frame [2]. Both ends of the untranslated regions (UTR) are highly conserved among all virus isolates, which also share high similarity with BVDV and BDV. These characteristics pose difficulties in performing conventional method such as fluorescent antibody test (FAT) or antigen-ELISA to discriminate CSFV from BVDV and BDV, which leads to imbalanced specificity and sensitivity. TaqMan real-time PCR (TaqMan-qPCR) can overcome these problems to a certain extent with its characteristics of short amplicon and use of a specific hydrolysis probe. Or simultaneously, it is of importance that the real-time PCR method can accurately detect and quantify the nucleic acids of pathogen [3-5].

Until recently, there were a few studies that performed real dynamic quantification of virus load and little is known about the virus load in relation to disease progression. As an example, a study to detect CSFV in experimentally infected pigs via virus isolation, RT-PCR and real time PCR[6] was somewhat limited, only demonstrating the presence or absence of viral genome in a tonsil and nasal swab, but without any indication of viral load. In this study, we aimed to quantify the viral copy number, based on the relative viral colonization density technique for relative quantification of viral copies. β-actin (ACTB), an endogenous gene was simultaneously amplified with RNA of CSFV to normalize the differences in the amount of total RNA added to each reaction. Therefore, the virus copy numbers are expressed relative to the expression of β-actin in each sample, this allows for real dynamic quantification of the virus load. We monitored the replication of CSFV dynamically via real-time detection to the changes nucleic acids of CSFV to determine a correlation between the CSFV load in a wide range of tissues and disease progression in experimentally infected pigs.

## Results

### Specificity of primers

The melting curves are generated at the end of the TaqMan-qPCR by slowly increased reaction temperature from 60°C to 95°C. Two melting curves displayed a single specific peak with Tm values at 84.2°C and 82.3°C, respectively. This indicated a single product. This specificity was also demonstrated by the sequencing results of the real-time amplification.

### Standard curves of the TaqMan-qPCR

**Figure 1:**
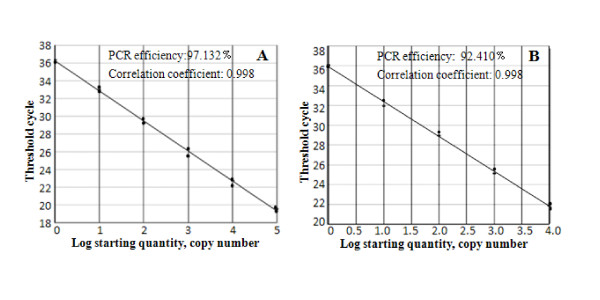
**Standard curves of the TaqMan-qPCR**. Ten serially diluted samples of genomic RNA from pigs infected with SM strain were used to develop the standard curves for CSFV (A) and ACTB (B).

Two typical standard curves were generated from the 10 serially diluted RNA amplification samples. The efficiency of the CSFV and ACTB amplification reactions were 97.132% and 92.410%, respectively. Both reactions showed good linear correlations (R^2 ^= 0.998) with a wide linear range CT ≤34 (Figure 1A and 1B).

### Specificity, sensitivity and repeatability of the TaqMan-qPCR

Excellent specificity and sensitivity were demonstrated by the positive results obtained for all 17 CSFV strains which contain the main genotypes in China (17/17) and the negative results for all 9 negative control samples (9/9). The detection limit of the TaqMan-qPCR was about 40 copies (5.3 × 10^-2 ^pg/μL) for CSFV RNAs and 52 copies (6.7 × 10^-2 ^pg/μL) for ACTB mRNAs.

The variation of CT values for each input RNA of five different strains between different runs was determined. The inter-assay and intra-assay coefficients of variation (CVs) for the detection of the five strains were equal or less than 2.80% (Table 1 and 2).

**Table 1:** Intra-assay performance of TaqMan qPCR with five different Chinese strains of CSFV

Samples	CT value									Result of intra-assay		
	
	1	2	3	4	5	6	7	8	9	Mean	SDs	CVs (%)
BJ1/08	21.23	21.39	21.42	21.43	21.53	21.60	23.10	23.14	23.17	21.67	0.366	1.62
BJ2/08	18.01	18.09	18.16	18.17	18.19	18.55	18.56	18.79	18.89	18.38	0.324	1.76
HeNXH2/98	21.11	21.14	21.17	21.40	21.44	23.02	23.17	23.28	23.55	21.70	0.557	2.45
FJFQ1/98	15.99	16.06	16.15	16.19	16.21	16.35	16.16	17.24	17.25	17.47	0.489	2.80
HeNBY1/96	24.70	25.04	25.06	25.41	25.05	25.81	25.82	26.29	26.39	26.42	0.599	2.27

9 repeats assay performed at the same time and under the same conditions

SD = standard deviation

CV = coefficient of variation

**Table 2:** The CT value and coefficient variation (CV) inter-assay of five samples

Samples	CT value									Result of inter-assay		
	
	1	2	3	4	5	6	7	8	9	Mean	SDs	CVs (%)
HCLV	21.50	21.77	21.19	20.10	20.23	20.14	20.27	20.67	20.03	20.88	1.024	4.91
JL5/99	23.45	24.51	24.59	23.05	23.52	23.41	23.54	24.20	23.21	23.72	0.565	2.38
HeBHH1/95	21.16	23.23	21.55	21.45	21.91	21.81	21.28	21.99	20.04	21.05	0.940	4.26
USA-331	16.41	17.85	17.07	15.94	16.31	16.16	15.85	16.10	15.69	16.38	0.682	4.16
GDGZ1/95	21.71	21.13	21.30	20.68	21.09	20.97	20.45	20.64	20.23	21.13	0.746	3.53

9 repeats assay performed at different times but under the same conditions

SD = standard deviation

CV = coefficient of variation

### Quantitative detection of virus load in the experimentally infected pigs

**Figure 2:**
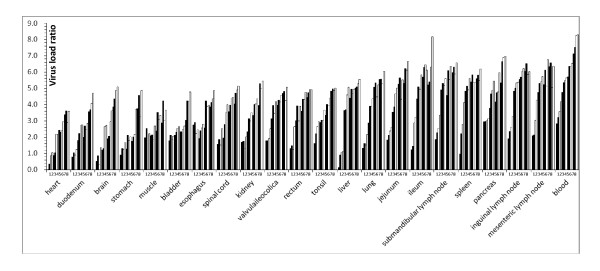
**Dynamic distribution of CSFV in the internal organs and blood of experimentally infected pigs**. As measured by TaqMan qPCR. All histograms are arranged according to the time after infection (i.e., Day 1 to 8). The viral load progressively increased over the 8 days.

Twenty-one internal organs and blood were obtained from the experimentally infected pigs and viral loads were quantified by TaqMan-qPCR at day 1-8 post infection. Then, series of CT values for CSFV and ACTB were obtained and normalized CT values (ΔCT) were determined. One of the skeletal muscle samples on day 2 generated the lowest ΔCT value and this was used to calibrate the data. Therefore, the calibrated quantification ratios were assessed by 2^-ΔΔCT^. To minimize inter-sample variation, the calibrated quantification levels were converted into logarithmic values and the results are shown in Figure 2.

The final data can be explained by two aspects. As shown in Figure 2, viral RNA could be detected in all the samples of the infected pigs from day 1 post infection and reached a peak at day 8 when the pigs were close to agonal stage. The viral load progressively increased over the 8 days.

**Figure 3:**
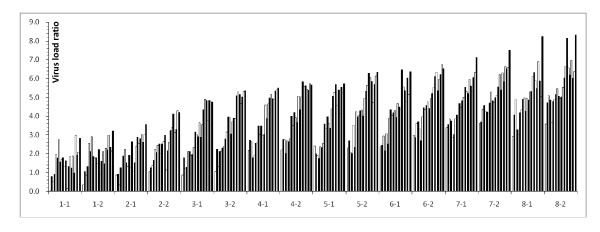
**The tissue tropism for 16 pigs challenged with the SM strain**. The histograms are arranged (from left) in the following sequence: heart, duodenum, brain, stomach, skeletal muscle, bladder, esophagus, spinal cord, kidney, valvula ileocolica, rectum, tonsil, liver, lung, jejunum, ileum, submandibular lymph node, spleen, pancreas, inguinal lymph node, mesenteric lymph node and blood. "1-1" and "1-2" represent pig1 and 2, respectively, on Day 1 post infection. The other code names are defined in a similar way.

As shown in Figure 3, the general trend of tissue tropism for the 21 internal organs and blood samples taken from each of the 16 pigs according to the viral load (from low to high) was as follows: heart, duodenum, brain, stomach, skeletal muscle, bladder, esophagus, spinal cord, kidney, ileocaecal valve, rectun, tonsil, liver, lung, jejunum, ileum, submandibular lymph node, spleen, pancreas, inguinal lymph node, mesenteric lymph node and blood. Except for the first 2 days post infection, the trends of tissue tropism remained consistent with that of the general trend from day 3 to day 8 post infection.

### Body temperature and clinical score

**Figure 4:**
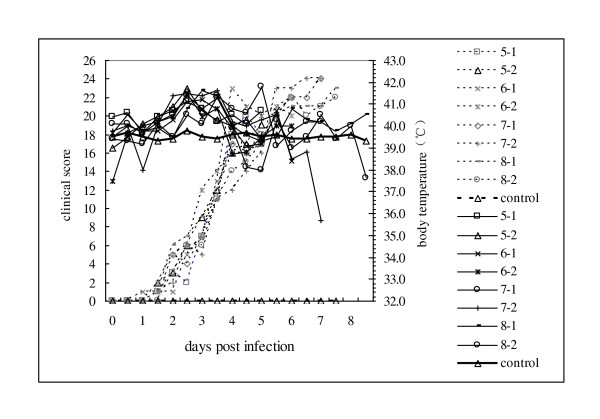
**Clinical score values and body temperature from CSF SM strain experimentally infected pigs**. The clinical score of the infected pigs showed a continued increasing trend from day 0 to day 8 post infection, which was found to correlate well with the virus load ratio. The noninfected control kept at a score of 0. The body temperature began to increase on the 2th day after infection. Then the temperature were kept around continued 41.5°C for 1 to 1.5 days, then began to decline at day 4 to 5 post infection. However, it showed irregular changes from day 6 to day 8 near to death. While the body temperature of the control group maintained at the normal level of 39.5°C.

All the 18 Pigs were examined daily by clinical inspection for signs of CSF, and body temperature determination. The average clinical score from day 1-7 are 0.56, 4.38, 9.81, 16.88, 19.07, 21.70, 22.50 respectively. Details were shown in Figure 4. (just presented day5-8) There was consistent relationship between clinical score and viral load ratio as shown in Figure 3.

### IHC staining of the experimentally infected pigs

**Figure 5:**
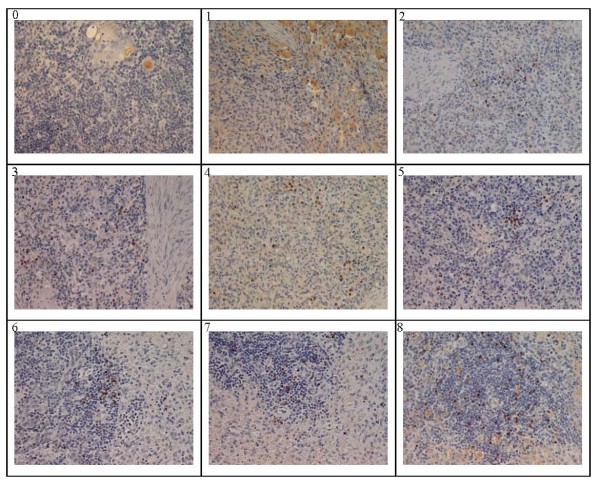
**CSFV antigen localization based on WH303-mediated immune-histochemistry for spleen from day 1 to 8**. IHC stains for CSFV in spleen of infection pigs euthanized form day 1 to day 8 post infection. The cells positive for viral antigens appeared dark-brown. Picture 0 was the negative control and 1, 2, 3, 4, 5, 6, 7 and 8 were day 1 to day 8 post infection respectively. The CSFV antigen positive cells were distributed in the medulla, cortex, dendritic reticulum and follicular epithelium. Magnification 400×.

Specific IHC was observed in 20 internal organs tested and the positive signal showed an increasing trend similar to the result of quantitative detection previously described from day 1-8 post infection as showed in Table 3. The results of the IHC staining showed in Figure 5 indicated that the CSFV antigen localization for the spleen based on WH303-mediated IHC from day 1 to 8 post infections. The positive signals appeared firstly in capillaries and then gradually transfer into the lymphocyte with an increasing trend. Meanwhile, none positive IHC signals appeared in the negative control.

**Table 3:** The results of the IHC staining in different tissues of pigs inoculated with CSFV

DPI	1		2		3		4		5		6		7		8		N
	
**Pig NO**.	1-1	1-2	2-1	2-2	3-1	3-2	4-1	4-2	5-1	5-2	6-1	6-2	7-1	7-2	8-1	8-2	
spleen	+++	+++	+++	+++	+++	+++	+++	+++	+++	+++	+++	+++	+++	+++	+++	+++	-
lung	+	+	+	+	+	+	+	+	+	+	+	+	+	+	+	+	-
liver	+	+	++	++	+++	+++	+++	+++	+++	+++	+++	+++	+++	+++	+++	+++	-
Ln1	++	-	+++	+++	+++	+++	+++	+++	+++	+++	+++	+++	+++	+++	+++	+++	-
kidney	+	-	++	++	+++	+++	+++	+++	+++	+++	+++	+++	+++	+++	+++	+++	-
duodenum	+	-	ND	++	+++	+++	+++	+++	+++	+++	+++	+++	+++	+++	+++	+++	-
tonsil	-	-	++	++	+++	+++	+++	+++	+++	+++	+++	+++	+++	+++	+++	+++	-
Ln2	-	-	+++	+++	+++	+++	+++	+++	+++	+++	+++	+++	+++	+++	+++	+++	-
Ln3	-	-	+++	+++	+++	+++	+++	+++	+++	+++	+++	+++	+++	+++	+++	+++	-
ileum	-	-	+++	+++	+++	+++	+++	+++	+++	+++	+++	+++	+++	+++	+++	+++	-
jejunum	-	-	+++	+++	+++	+++	+++	+++	+++	+++	+++	+++	+++	+++	+++	+++	-
rectum	-	-	++	ND	+++	+++	+++	+++	+++	+++	+++	+++	+++	+++	+++	+++	-
stomach	-	-	-	+	+++	+++	+++	+++	+++	+++	+++	ND	ND	ND	ND	ND	-
bladder	-	-	-	+	ND	ND	++	-	ND	ND	++	+++	+++	+++	+++	+++	-
pancreas	-	-	-	++	+++	+++	+++	+++	+++	+++	+++	+++	+++	+++	+++	+++	-
brain	-	-	-	+	-	-	+	-	+	++	++	++	++	+++	+++	+++	-
spinal cord	-	-	-	+	-	-	+	+	-	+	+	+	+	+	+	+	-
ileocecal valve	-	-	ND	ND	+++	ND	+++	ND	+++	ND	+++	+++	+++	+++	+++	+++	-
skeletal muscle	-	-	-	-	-	-	++	-	++	++	++	++	++	++	++	++	-
heart	-	-	-	-	-	-	-	-	+++	+++	+++	+++	+++	+++	+++	+++	-

"-" = negative, "+" = 1-3 foci/section "++" = 4-10 foci/section "+++" = >10 foci/section

Ln1 = Submandibular lymph node

Ln2 = Mesenteric lymph node

Ln3 = Inguinal lymph nodes

N = Negative control

ND = Not done

## Discussion

CSF is a severe swine disease in China [7], resulting in substantial economic loss. However, we are not fully understand the viral pathogenesis, multiplication, distribution, tissue tropism and excretion in pigs. In this study, TaqMan-qPCR was used to detect the viral RNAs in various internal organs, including heart, duodenum, brain, stomach, skeletal muscle, bladder, oesophagus, spinal cord, kidney, ileocaecal valve, rectum, tonsil, liver, lung, jejunum, ileum, submandibular lymph node, spleen, pancreas, inguinal lymph node, mesenteric lymph node and blood at different time points p.i. Using these experimentally infected pigs as a model, we determined the dynamic distribution and the viral tissue tropism. At the same time, an E2 monoclonal antibody WH303-based IHC test was used to verify the quantitative results and the same trend were observed.

The virus load of CSF was almost highest in lymphoid tissue and blood. Interestingly, we found that the ileum and the pancreas showed high viral colonization density. Meanwhile, the heart, duodenum, brain, stomach and skeletal muscle had relatively low viral content. It is surprising that the tonsil, which is anticipated to contain the most abundant viral load, had only moderate viral content.

Many researchers have done similar studies using different strains and different target tissues [8-12]. Previous studies have shown that the CSFV antigen was not detected in heart tissue at any time and that the highest levels of antigen detection were found in the tonsils, spleen and pancreas using IHC test [8]. Considering these findings, we can explain these differences as follows. First, The clinical signs of CSF are extremely variable and it may be confused with many other diseases. The age of the animal, virus virulence and dose lead to distribution differences among acute, chronic and prenatal forms [13]. It may be possible to detect positives for highly virulent strain and high dose groups earlier than those of lower virulence strains or doses because of more rapid transport to the tissues and higher viral load delivered. Second, the detection methods such as virus isolation, AC-ELISA [14], in-situ hybridization and IHC [15], RT-PCR, RT-nPCR [16,17] and real-time PCR [3,18] have different sensitivities. Real-time PCR is believed to be more sensitive than the other assays. Third, we collected samples daily from day 1 to day 8 post infections, when the pigs were close to agonal stage, which is different from other models, and may be responsible for distribution differences. Other sample collection times may miss the viral multiplication peak.

Here, we determined the viral load in 21 different organs and blood, including the digestive system, respiratory system, circulatory system and nervous system. This provided us with larger samples so that we could obtain better understanding about the distribution of viral infection.

Relative quantitative RT-PCR, as described by Livak and Pfaffl [19,20] and termed the Livak model and the Pfaffl model, respectively, is widely used to quantify mRNA transcription [21-23]. However, in this study, the dynamic distribution was determined using colonization density as the final determination index. This technique can be applied to assess the relative viral expression to mRNA expression within tissues. Our study is one of the first to use this approach. This concept was recently reported for *infectious bursal disease virus *[24]. To achieve this goal, an internal control gene (ACTB), which has the highest stability across tissues with consistent expression, was needed [25] to normalize the initial RNA levels between different samples. Selection of high-quality reference genes is of great importance for the interpretation of data generated by real-time qPCR[26]. In our experiment, the CSFV and ACTB were optimized to have equal amplification efficiency, which is suitable for the Livak model, and it provided reliable results. Recently, some studies have showed that it is better to use multiple internal reference genes for relative quantification [27]. Nevertheless, we only used ACTB as an endogenous gene; if we added another gene such as GAPDH, 18sRNA or HPRT as complementary internal control, this may have provided different results. We consider that, despite the different internal control, the final results will not be greatly affected for the following reasons. First, all the candidate reference gene are expressed at constant levels relative to viral amplification. Second, the copy number of reference genes per cell is much less than the RNA copy numbers of the infected virus.

## Conclusions

In conclusion, the viral RNA could be detected in all the samples of the infected pigs from day 1 post infection and reach a peak at day 8 when the pigs were close to agonal stage. The viral load progressively increased over the 8 days, which matched well with the clinical score and IHC result. The general trend of tissue tropism for the 21 tissue samples and blood (from low to high) was as follows: heart, duodenum, brain, stomach, skeletal muscle, bladder, esophagus, spinal cord, kidney, ileocaecal valve, rectum, tonsil, liver, lung, jejunum, ileum, submandibular lymph node, spleen, pancreas, inguinal lymph node, mesenteric lymph node and blood.

Our results provide significant data for further clarification of the pathogenic mechanism of CSFV in internal organs and further studies should be performed to determine the potential receptors in organs such as lymphoid tissue, ileum and pancreas. It is of importance to do similar studies in cases of chronic infection.

## Methods

### Primers and probes

Text for two specific oligonucleotide primers and one fluorogenic probe were designed based on the genomic sequences of 34 CSFV strains and five BVDV strains published in GenBank. Both primers and the probe were designed to target a highly conserved region within the 5' UTR of the CSFV genome. The locations and sequences of the primers and probes were as follows: forward primer, CSFV-F, starting at base position 172, 5'-TAC AGG ACA GTC GTC AGT AGT TCG A-3'; reverse primer, CSFV-R, starting at base 246, 5'-CCG CTA GGG TTA AGG TGT GTC T-3'; and probe, CSFV-P, starting at base 209, 5'-CCC ACC TCG AGA TGC TAT GTG GAC GA-3'. The TaqMan probe was labeled with a 5' reporter dye, FAM (6-carboxyfluorescein), and a 3' quencher, TAMRA (5carboxytetramethylrhodamine).

The two primers and the probe for ACTB were designed based on the mRNA sequence of ACTB published in GenBank (AK237086.1) as follows: forward, ACTB-F: 5'-CTC CGA TCT GTG CAG GGT ATT-3'; reverse, ACTB-R: 5'-CCC GCA AGA CAG AAA TGA CAA-3'; probe, ACTB-P: 5'-TGT GTC CGA GCT CCT ATT CCA GGA TTT CTC-3'. The probe was also labeled with a 5' reporter dye, FAM, and a 3' quencher, TAMRA.

### Viruses and bacteria

The CSFV SM (F114, 1998.6.28), HCLV (F479, 1996.3.28), Thiverval strain, BVDV Oregon C24V, NADL strain, the *Porcine parvovirus *(PPV)09/79 strain and *Foot and mouth virus *(FMDV) C2101 strain, *E. coli *(79-3A), *S. aureus *(C56023) and *S. choleraesuis *(C78-4) were provided by the China Veterinary Culture Collcetion Center (CVCC). The HeNBY1/96, HeBHH1/95, HeNXH2/98, BJTX3/96, GDGZ1/95, BJCY1/96, HeBZJK1/99, JL1/94, JL5/99, GXBH1/98, SZGM1/98, BJSY1/01, FJFQ/98 and BJTX1/95 strains were isolated and maintained by the National Classical Swine Fever Reference Laboratory, China. The above mentioned strains are the main subgenotypes(1.1/2.1/2.2)in China. *Porcine reproductive and respiratory syndrome virus *(PRRSV) BJ strain was provided by the Preventive Veterinary Medicine's Key Opening Laboratory of Ministry of Agriculture, China. *Pseudorabies virus *(PRV) BJ/99, Fa strain was provided by Sichuan Agricultural University, China.

### Experimental animals

Eighteen 15-20 kg out-bred white weaned pigs were used in this study. They were born from non-immune sows and tested as sera negative for CSFV antibodies and CSFV antigen. Eighteen weaned pigs were tested for both CSFV antibodies and antigens and were negative for both. In addition, eighteen weaned pigs were tested for antibodies and antigens of BVDV, PRRSV, PCV-2, PPV and PRV and were negative all. They were then divided into experimental group (16 pigs) and control group (2 pigs) and were housed separately in different buildings following strict biosafety measures to prevent cross-infection.

The experimental group were given an intramuscular injection with 1 ml of blood containing 4 × 10^3.84^TCID_50 _(50% tissue culture infection dose) SM virus. Two experimental pigs were killed randomly by electrocution every day between days 1 and 8. 21 organs and blood were collected from each pig and were analysed by the TaqMan-qPCR and IHC test. The control group comprised two pigs, which was injected with the same volume of PBS. Blood samples were collected daily until day 8 when two pigs were killed. 21 organs and blood were also collected and tested as described in the experimental group.

Clinical signs of CSF were evaluated by using a score system suggested previously [28] in order to follow the progression of the disease. Body temperature was recorded daily.

### RNA and DNA Extraction

RNAs were extracted using TRIzol^® ^reagent (Invitrogen, USA) according to the manufacturer's instructions. DNAs were extracted using the saturated phenol-chloroform method. Total RNA and DNA purification and quantity were assessed by spectrophotometer analysis at 260 and 280 nm (Nano Drop^® ^ND-1000).

### Primers test with RT-PCR and SYBR Green I melting curve analysis

Conventional RT-PCR was carried out with CSFV and ACTB primers; then the PCR products were electrophoresed on a 1.5% agarose gel and sequenced to validate the products.

The two pairs of primers were detected by real-time PCR melting curve analysis using SYBR Premix Ex Taq™ kit (TaKaRa) according to the manufacturer's instructions to verify the specificity of the amplification product.

### TaqMan-qPCR

The CSFV amplification system contained the following components: 5 μL of 10 × PCR buffer (10 mM pH 8.3 Tris-HCl, 50 mM KCl, 1.5 mM MgCl_2_), 1.5 μL of dNTP mixture (2.5 mM), 100 U of SSIII reverse transcriptase (Invitrogen), 2.5 U of TaqHS DNA polymerase (TaKaRa), 3 μL each of the primers CSFV-F and CSFV-R (30 μM), 1.5 μL of CSFV-P (7.5 μM), 10 μL of RNA solution. DEPC water was added to a final volume of 50 μL. The ACTB amplification system contained the following components: 5 μL of 10 × PCR buffer (10 mM pH 8.3 Tris-HCl, 50 mM KCl, 1.5 mM MgCl_2_), 1.5 μL of dNTP mixture (2.5 mM), 100 U of SSIII reverse transcriptase (Invitrogen), 2.5 U of TaqHS DNA polymerase (TaKaRa), 2 μL of ACTB-F (20 μM), 3 μL of ACTB-R (30 μM), and 0.8 μL of ACTB-P (4 μM), 10 μL of RNA solution. DEPC water was added to 50 μL.

10 serially diluted genomic RNA samples of the SM strain used to infect the pigs were amplified using the above two optimized reaction systems, and standard curves were formed by plotting the CT values against the log concentration of the initial RNA with the CT (threshold cycle) values at the range of 15~35. The efficiency of each reaction was determined by calculating the slopes of the standard curves generated in Figure 1a and 1b using the equation E% = (10^(-1/slope)-1) × 100%.

The thermal conditions were 50°C reverse transcription for 20 min; initially denaturation at 94°C for 4 min; followed by 40 cycles of denaturation at 88°C for 8 seconds, annealing and extension were combined into one step at 60°C for 35 secconds, and the fluorescent signal was measured at 60°C.

### Specificity, sensitivity and repeatability test

The TaqMan-qPCR method was used to detect the nucleic acid of the 17 CSFV strains mentioned above and another 11 related pathogenic viruses and bacteria in pigs were used as negative controls to determine specificity of the procedure. 10 serially diluted genomic RNA of the SM strain was assayed by the TaqMan-qPCR to determinate its sensitivity.

Inter-assay and intra-assay reproducibility tests were detected in triplicates by testing five different strains to evaluate the reproducibility of the TaqMan-qPCR assay.

### Detection of CSFV in experimentally infected pigs by TaqMan-qPCR

Total RNAs were extracted from tissues and organs of the experimentally infected pigs. Then the same volume of the total RNA (10 μL) was added in triplicate to the two amplification systems. One-step real-time TaqMan-qPCR in a reaction volume of 50 μL was carried out in an ABI7500 instrument. The RNAs of CSFV and ACTB were simultaneously amplified in one plate and shared the same optimized thermal condition described above. Therefore, we obtained three CT values for both CSFV and ACTB for each sample. Average of these CT values (CT_CSFV_) and (CT_ACTB_) were used to determine the relative colonization density using the 2^-ΔΔCT ^method. ACTB was used as an internal reference to normalize the data.

### Detection of CSFV in experimentally infected pigs by Immunohistochemistry

In this study, monoclonal antibody WH303 was kindly provided by Prof. Trevor Drew of Veterinary Lab-oratory Agencies, Addlestone, UK. The monoclonal antibody was specific to CSFV E2 protein.

After euthanasia, spleen, lung, lever, submandibular lymph nodes, kidney, duodenum, tonsil, mesenteric lymph node, inguinal lymph nodes, ileum, jejunum, rectum, thymus, stomach, abdominal salivary gland, brain, spinal cord, ileocecal valve, urinary bladder, skeletal muscle, cardiac muscle and blood of infected pigs were collected and fixed in 4% paraformaldehyde, embedded in paraffin wax and sectioned at 4 μm thickness. The paraffin-embedded sections were dewaxed in the xylene and rehydrated through graded decreased alcohols into PBS. And then those tissue sections were incubated with 3% (v/v) hydrogen peroxide to inactivate the endogenous peroxidase for 20 minutes. The tissue sections were rinsed twice with PBST (0.1 M PBS containing 0.05% Tween-20, pH 7.4) and submitted to antigen retrieval in citrate buffer solution (0.01 M, pH 6.0) by microwaving for 20 min. After cool down to room temperatures, the tissue sections were incubated with 1% normal horse serum for 30 minutes at room temperature to block the non-specfic binding sites and then rinsed the tissue sections thoroughly with PBST. The monoclonal antibody WH303 was diluted 1:200 with PSBT containing 1% BSA. The tissue sections were covered with the diluted monoclonal antibody at room temperature for 1 hour, meanwhile negative controls were incubated with PBST containing 2% BSA instead of primary specific monoclonal antibody as negative controls. After rinsed three times with PBST, the sections were covered with secondary antibody, peroxidase conjugated rabbit anti mouse antibody, diluted 1:100 with PBST containing 1% BSA and incubated for 20 minutes at room temperature. Then the sections were rinsed with PSBT for three times and stained with 0.05% (v/v) 3, 3'-diaminobenzidine tetrahydrochloride (DAB, Sigma) containing 0.03%(v/v) hydrogen peroxide in Tris- HCl buffer (0.05 M Tris-HCl, 0.15 M NaCl, PH7.6) for 5 minutes. Finally, the sections were rinsed with tap water, counterstained with hematoxylin, dehydrated in graded alcohol, cleaned in xylene and then mounted with DPX (Sigma) by turns.

### Data analysis

Quantitative real-time PCR data were processed with SDS 2.0 software package (ABI7500). Samples with a CT value >34 were excluded from the analysis and the comparisons were performed only for samples with a CT value ≤34. Linear regression analysis was used to compare the colonization density data (ΔCT) from samples (ΔCT _sample _= CT_CSFV sample _- CT_ACTB sample_) *vs *calibration (ΔCT_calibration _= CT_CSFV calibration _- CT_ACTB calibration_). The relative colonization density was determined as the ΔΔCT between samples and calibration (ΔΔCT = ΔCT_sample _- ΔCT_calibration_), and the relative quantification of the virus load was assessed by the 2^-ΔΔCT ^method.

## Competing interests

The authors declare that they have no competing interests.

## Authors' contributions

QW and QZZ conceived and supervised the study, also helped to draft the manuscript. JL and XZF performed the main experiments and draft the manuscript., LX, XQZ, WH and SS participated in the animal experiments. BT, LC and YYZ participated in the animal experiments and carried out the molecular genetic studies. YCZ carried out the immunohistochemistry. All authors read and approved the final manuscript.
